# sEMG-Based Gain-Tuned Compliance Control for the Lower Limb Rehabilitation Robot during Passive Training

**DOI:** 10.3390/s22207890

**Published:** 2022-10-17

**Authors:** Junjie Tian, Hongbo Wang, Siyuan Zheng, Yuansheng Ning, Xingchao Zhang, Jianye Niu, Luige Vladareanu

**Affiliations:** 1 Parallel Robot and Mechatronic System Laboratory of Hebei Province, Yanshan University, Qinhuangdao 066004, China; 2 Academy for Engineering & Technology, Fudan University, Shanghai 200433, China; 3 Institute of Solid Mechanics of the Romanian Academy, 010141 Bucharest, Romania

**Keywords:** sEMG, lower limb rehabilitation robot, compliance control, training mode, MOTOmed, continuous passive motion, straight leg raise, feature analysis

## Abstract

The lower limb rehabilitation robot is a typical man-machine coupling system. Aiming at the problems of insufficient physiological information and unsatisfactory safety performance in the compliance control strategy for the lower limb rehabilitation robot during passive training, this study developed a surface electromyography-based gain-tuned compliance control (EGCC) strategy for the lower limb rehabilitation robot. First, the mapping function relationship between the normalized surface electromyography (sEMG) signal and the gain parameter was established and an overall EGCC strategy proposed. Next, the EGCC strategy without sEMG information was simulated and analyzed. The effects of the impedance control parameters on the position correction amount were studied, and the change rules of the robot end trajectory, man-machine contact force, and position correction amount analyzed in different training modes. Then, the sEMG signal acquisition and feature analysis of target muscle groups under different training modes were carried out. Finally, based on the lower limb rehabilitation robot control system, the influence of normalized sEMG threshold on the robot end trajectory and gain parameters under different training modes was experimentally studied. The simulation and experimental results show that the adoption of the EGCC strategy can significantly enhance the compliance of the robot end-effector by detecting the sEMG signal and improve the safety of the robot in different training modes, indicating the EGCC strategy has good application prospects in the rehabilitation robot field.

## 1. Introduction

Lower limb motor dysfunction is a common sequela of stroke patients. The elderly is a high-risk group for stroke, and as the population ages, the incidence of stroke increases dramatically [1,2]. The plasticity of the human brain and central nervous system is the basis of rehabilitation medicine. Through the training exercise of specific tasks and the use of the motor relearning program of the nervous system, the motor function of the patient’s lower limbs can be effectively restored [3,4,5]. Rehabilitation robotics, as an emerging technology developed in the rehabilitation field, has advantages in clinical and biomechanical measurements compared with conventional therapy [6]. In addition, the rehabilitation robot is relatively easy to manage and control, which can help patients perform predetermined training actions accurately and repeatedly and improve the effectiveness of rehabilitation treatment [7]. In recent years, the design and control strategies of rehabilitation robots have become research hotspots in the fields of rehabilitation engineering and robotics.

With the development of robotics and rehabilitation theory, various lower limb rehabilitation robots have been designed. Lower limb rehabilitation robots are mainly divided into exoskeleton type and end-effector type [8]. In the exoskeleton robot system, there is a one-to-one correspondence between the robot and human joints. The exoskeleton robot system can be worn on the human body and usually has a compact structure [9]. The lower limb exoskeleton robot MotionMaker adopts the integrated design of the seat and lower limb motion mechanism, which can carry out passive, semi-active, and active training modes [10]. Li et al. designed a lower limb exoskeleton rehabilitation robot which can assist the patient in carrying out gait training [11]. Feng et al. designed a lower limb rehabilitation robot for passive training of stroke patients, and the moving seat can be adjusted or separated from the robot to meet the rehabilitation demands of patients at different stages [12]. Akdoğan et al. produced a therapeutic exercise robot Physiotherabot, which can perform active and passive movements and learn specific exercise movements [13]. In the end-effector robot system, pedals or platforms are used to generate limb motion from the distal end of the lower limb without requiring alignment between the robot and human joints. Wang et al. designed a rigid-flexible end-effector lower limb rehabilitation robot, which consists of a rigid mobile device and a flexible drive system, which can realize the adduction/abduction and internal/external rotation movement of the lower limb [14]. Bouri et al. developed a parallel robot Lambda that can be used to guide the movement of the lower limb and carry out rehabilitation training of the hip, knee, and ankle joints [15]. Saglia et al. developed a 3-UPS/U parallel mechanism, which can perform rehabilitation training of the human ankle joint [16].

According to the active participation degree of patients, rehabilitation training can be divided into three categories: passive training, semi-active training, and active training [17]. In the passive training process, the rehabilitation robot guides the affected limb to move along a predetermined trajectory for rehabilitation training [18]. For the passive training modes of lower limb rehabilitation robots, the typical ones include MOTOmed training mode, continuous passive motion (CPM) training mode, and straight leg raise (SLR) training mode [19,20,21,22]. In the MOTOmed training mode, the end trajectory of the robot is a circular trajectory; In the CPM training mode, the end trajectory of the robot is a linear trajectory; In the SLR training mode, the end trajectory of the robot is an arc trajectory. In order to improve the safety and comfort of patients during passive rehabilitation training, numerous studies have been conducted on the compliance control strategy of the lower limb rehabilitation robot. Wang et al. [23] proposed a fuzzy sliding mode variable admittance controller based on safety evaluation and supervision for the cable-driven lower limb rehabilitation robot, which can switch between active training mode and passive training mode and adjust the parameters of the admittance controller. Li et al. [24] designed a multi-modal control scheme for exoskeleton rehabilitation robots, including robot-assisted mode, robot-dominant mode, and safety-stop mode, and verified the effectiveness of the scheme in upper-limb and lower-limb exoskeleton robot systems. Zhou et al. [25] proposed a trajectory deformation algorithm, which can realize the desired trajectory planning of participants based on the interaction force in the process of human-robot interaction and improve robot compliance and motion smoothness. Chen et al. proposed a reference trajectory adaptive compliance control algorithm, which combines impedance control and motion trajectory planning [26]. Huo et al. developed a lower limb exoskeleton impedance modulation strategy, which can provide proper power and balance assistance during sit-to-stand movements [27]. Compared with the position control strategy, the compliance control strategy is beneficial in avoiding excessive force between the human and the robot and has a wider application in the field of rehabilitation robots [28].

The sEMG-based control strategies of the lower limb rehabilitation robot mainly include the sEMG-based continuous control strategy and the sEMG-triggered control strategy [29]. Many studies have been carried out on the sEMG-based continuous control strategy, in which the lower limb motion intention recognition is performed using the sEMG signal and torque assistance proportional to the sEMG signals is provided to generate the desired motions. Khoshdel et al. proposed an sEMG-based robust impedance control strategy for the lower limb rehabilitation robots and the sEMG signals were used to estimate the exerted force [30]. Yao et al. developed an adaptive admittance control scheme consisting of an admittance filter, an inner position controller, and an sEMG-driven musculoskeletal model [31]. Xie et al. proposed an adaptive trajectory planning method based on sEMG signals and interactive forces for lower limb rehabilitation robots and planned three periodic trajectories using sEMG signals [32]. Different from the sEMG-based continuous control, the robot assistance is triggered when the sEMG signals reach a certain threshold in the sEMG-triggered control strategy. Meng et al. proposed an active interactive controller based on motion recognition and adaptive impedance control. Using the root mean square (RMS) feature of the sEMG signal integrated with the support vector machine (SVM) classifier, it can predict the motion intention of the lower limbs and trigger robot assistance [33]. Lin et al. designed an sEMG-triggered controller for the artificial muscle-driven lower limb rehabilitation robot, and the methods of discrete wavelet transformation and the support vector machine are used to predict the lower limb movement intention [34]. Compared with force and position signals, the sEMG signals can reflect the activity level of specific muscle groups, which can monitor and control the movement of limbs in more detail [35].

However, the above-mentioned compliance control strategies for lower limb rehabilitation robots using sEMG signals are mainly aimed at active training scenarios. Existing passive training control strategies mainly rely on force and position information and lack the intelligent sEMG-based compliance adjustment function, resulting in an unsatisfactory safety performance of lower limb rehabilitation robots [36]. Moreover, in the passive training process of lower limbs, the essential purpose of adopting different training modes is to perform specific training effects on different muscle groups. The fusion of the force, position and sEMG signals in the compliance control strategy, monitoring the muscle activation degree in real time, and controlling the motion of the robot, encompass a significant problem to be solved in the control strategy development of the lower limb rehabilitation robot [36].

Aiming at the problems above, based on the hybrid end-effector lower limb rehabilitation robot (HE-LRR) developed in our research group [37], this paper proposes an sEMG-based gain-tuned compliance control (EGCC) strategy. In the passive training process, the lower limbs follow the robot end effector to move in three-dimensional space. The human body keeps the lower limbs relaxed and does not actively contract muscles. The sEMG signal collected under this condition is intended to monitor the muscle condition and protect the patient by enhancing robot compliance. The rationality of the control strategy is verified through simulation and experimental research under three training modes: MOTOmed, CPM, and SLR. The rest of this paper is organized as follows. Section 2 contains the introduction of the configuration design of the HE-LRR. The EGCC strategy is proposed in Section 3. The simulation research of the EGCC strategy without sEMG information is performed in Section 4. In Section 5, the sEMG acquisition and feature analysis are carried out, and the EGCC strategy comprehensive experiment is conducted. Section 6 presents the conclusions and prospects for the EGCC strategy.

## 2. Robot Configuration

**Figure 1:**
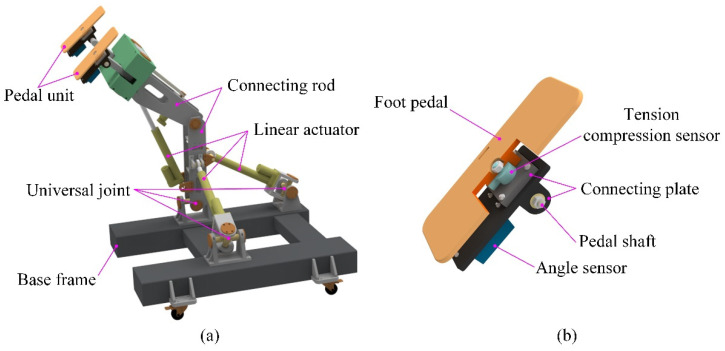
(**a**) The virtual prototype of the HE-LRR; (**b**) structure of pedal unit.

There are mainly three types of lower limb movement for the human body, namely moving in the sagittal plane, stepping in the coronal plane, and turning around the longitudinal axis of the human body [38]. HE-LRR is designed in accordance with ergonomic considerations, which includes a base frame, a hybrid (2UPS+U)&(R+RPR) mechanism, and a pedal unit. Here U, P, R, and S represent a universal pair, a prismatic pair, a revolute pair, and a spherical pair, respectively. Figure 1 shows the virtual prototype of the HE- LRR and the pedal unit. The HE-LRR allows people to sit or lie on the opposite side of the machine while their feet are connected to the robot end effector, and they receive rehabilitation training.

According to the simplified rotation characteristics of the hip joint where two rotation axes are orthogonal, the parallel part of the lower limb rehabilitation robot is designed as a (2-UPS+U) mechanism, including two UPS branches and one U branch chain. Using linear actuators, the parallel part is driven to rotate around the cross axis, thereby assisting the lower limbs in achieving rehabilitation training in the sagittal and coronal planes. In order to realize the rotary motion of the knee joint, the RPR branch chain is introduced into the parallel part, and the linear actuator is used as the driving unit. Rehabilitation training requirements for patients with multiple degrees of freedom can be met by the coordinated movements of (2-UPS+U)&(R+RPR) mechanisms. The (R+RPR) mechanism is superior to rotary motor driving, and it can reduce the mass and inertia of the kinematic joint of the robot and increase its bearing capacity.

The pedal unit is composed of a foot pedal, a pedal shaft, connecting plates, a tension compression sensor, and an angle sensor. The foot pedal is utilized to guide the distal end of the lower limb to move while the pedal shaft is used to connect the pedal unit with the hybrid mechanism. The tension compression sensor is embedded in the pedal unit to record the man-machine contact force, and the angle force is installed on the connecting plate to acquire the angle information of the pedal unit.

## 3. EGCC Strategy

There are two typical impedance control strategies applied in rehabilitation robots: the force-based impedance control strategy and the position-based impedance control strategy. Although the force-based impedance control strategy can realize force tracking, the controller relies on the dynamic characteristics between the robot and the environment, making it difficult to implement control in practice. Compared with the force-based impedance control, the position-based impedance control has more stable performance [39,40]. In this section, the passive training of the lower limb rehabilitation robot adopts a position-based impedance control strategy. The impedance control model is as follows:

(1) $$M_{d}\Delta \ddot{X}+B_{d}\Delta \dot{X}+K_{d}\Delta X=F$$

where, *M*_d_, *B*_d_, *K*_d_ are the target inertia matrix, damping matrix, and stiffness matrix of the impedance model; *F* is the man-machine contact force acting on the robot end effector; Δ*X* is the position correction amount of the robot end effector.

Using Laplace transformation, the position correction amount in the Laplace domain can be derived as follows:

(2) $$\Delta X(s)=\frac{F(s)}{M_{d}s^{2}+B_{d}s+K_{d}}$$

where, *s* is the complex number frequency parameter.

**Figure 2:**
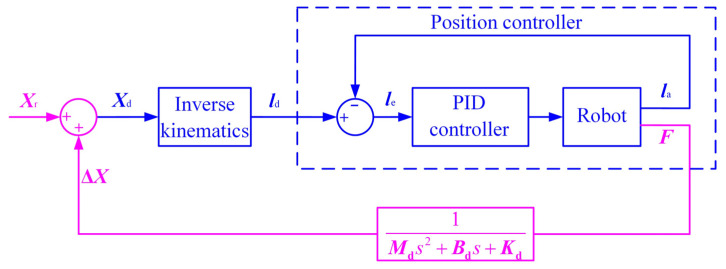
Position-based impedance control strategy diagram.

The block diagram of the position-based impedance control is shown in Figure 2. The man-machine contact force *F* passes through the impedance control model to generate the position correction amount Δ*X*, which is superimposed on the reference position *X*_r_ to generate the desired position *X*_d_, which is sent to the position controller after the inverse kinematics solution, so that the actual position tracks the desired position.

**Figure 3:**
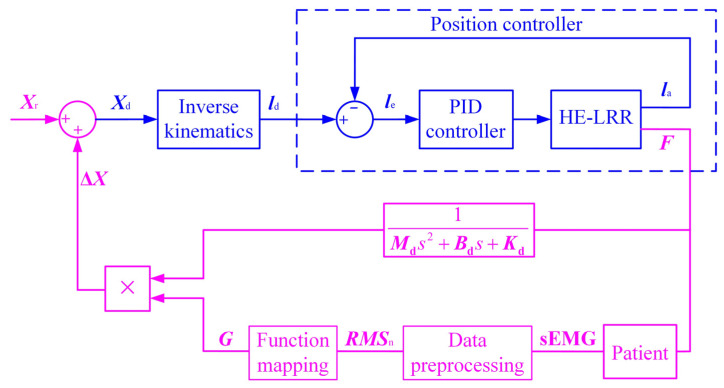
EGCC strategy diagram for the HE-LRR.

The above position-based impedance control strategy is suitable for not only controlling the robot to move along a preset trajectory, but also maintaining a certain flexibility during the movement. The method is to convert the end contact force into the position correction amount through the impedance control model. In order to improve the compliance and safety of the control strategy, the sEMG information needs to be integrated into the above position-based impedance control strategy. The modified EGCC strategy diagram is shown in Figure 3.

From the original sEMG signal of the patient’s target muscle group to the gain parameter, it needs to go through two processes: data preprocessing and function mapping. In the process of data preprocessing, the high-frequency and low-frequency signals are filtered out of the sEMG signal through the band-pass filter, and then the time-domain features with the intuitive physical significance are obtained through feature extraction. The root mean square (RMS) can reflect the average power of the sEMG signal, so the RMS feature value is used to evaluate the characteristics of the sEMG signal, and the calculation formula is as follows:

(3) $$RMS_{j}=\sqrt{\frac{1}{W}\sum_{i=1}^{W}x_{i}^{2}}$$

where *j* represents the *j*-th segment in the original sEMG data sequence, *x_i_* is the *i*-th original data in the segment data, and *W* is the sliding window width.

In order to improve the generalization ability of the model, the sEMG signals after feature extraction need to be normalized. The normalization calculation formula is as follows:

(4) $$RMS_{n}=\frac{RMS-RMS_{\min}}{RMS_{\max}-RMS_{\min}}$$

where *RMS* represents the sEMG signal after feature extraction; *RMS*_min_ and *RMS*_max_ are the minimum and maximum values of *RMS*, respectively; *RMS*_n_ is the normalized sEMG signal. *RMS*_min_ and *RMS*_max_ are constants in different training modes and can be obtained through sEMG signal acquisition and feature analysis (see Section 5.2). Here the gain parameter *G* is set to be 1, that is, the sEMG signal is not included in the control strategy during the sEMG acquisition experiment.

After the normalization processing, the normalized sEMG signals *RMS*_n_ of different muscle groups can be obtained according to Equation (4) respectively. In the “Function mapping” block, the maximum value of the muscle groups’ normalized sEMG signals is compared with the threshold value of the normalized sEMG signal *RMS*_t_, and the gain parameter *G* can be calculated according to the following equation:

(5) $$G=\{\begin{array}{cc} 1 & RMS_{n}\leq RMS_{t}\\ a{\left(RMS_{n}-RMS_{t}\right)}^{2}+1 & RMS_{n}>RMS_{t} \end{array}$$

When the normalized sEMG signal does not exceed the threshold value, the gain parameter is equal to 1. Otherwise, there is a quadratic functional relationship between the gain parameter and the normalized sEMG signal. Thus, in the passive training process of the lower limb rehabilitation robot, the position correction amount is jointly affected by the inertia parameter, damping parameter, stiffness parameter, and gain parameter. When the normalized sEMG threshold is constant, the maximum value of the gain parameter *G* is determined by the parameter *a*. If the parameter *a* is too large, the position correction amount will be too large, it will become more difficult for the robot end effector to move near the set trajectory, and the patient will not be able to receive standardized rehabilitation training. If the parameter *a* is too small, the position correction amount is too small, and the robot end effector will have no apparent sEMG-based compliance enhancement effect in the EGCC strategy. Therefore, the parameter *a* should be kept within a moderate range.

## 4. Simulation and Results

### 4.1. Impedance Control Parameter Influence Analysis

In the passive training process, it is important to select appropriate inertia parameters, damping parameters, and stiffness parameters when applying the impedance control model. Therefore, it is necessary to analyze the influence of impedance control parameters on the control performance. The transfer function of the impedance control model is:

(6) $$G(s)=\frac{\Delta X(s)}{F(s)}=\frac{1}{M_{d}s^{2}+B_{d}s+K_{d}}$$

For the convenience of analysis, considering the impedance control model in a single direction, Equation (6) can be simplified to Equation (7):

(7) $$G(s)=\frac{1}{ms^{2}+bs+k}$$

where, *m*, *b*, and *k* are the inertia parameter, damping parameter, and stiffness parameter, respectively. Equation (7) is transformed into the standard form:

(8) $$G(s)=\frac{1}{k}\frac{\omega_{n}^{2}}{s^{2}+2\xi \omega_{n}s+\omega_{n}^{2}}$$

where, *ω_n_* is the undamped natural frequency; *ξ* is the damping ratio.

**Figure 4:**
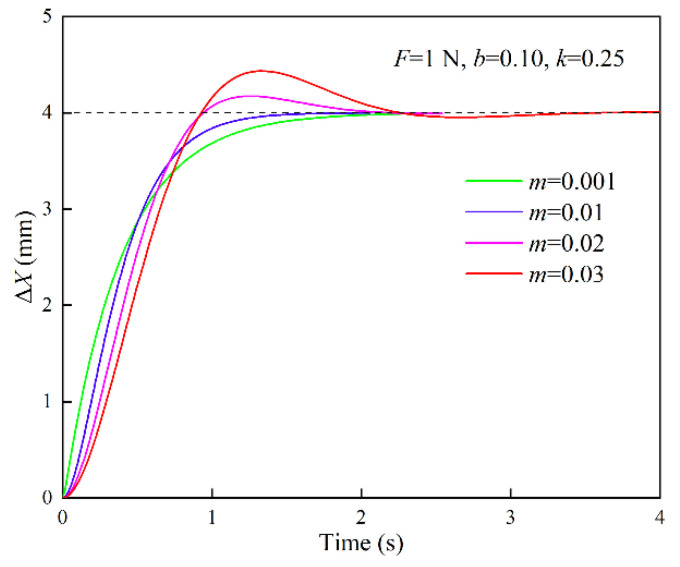
Response curves of position correction amount under different inertia parameters (*m*: N·s^2^/mm; *b*: N·s/mm; *k*: N/mm).

**Figure 5:**
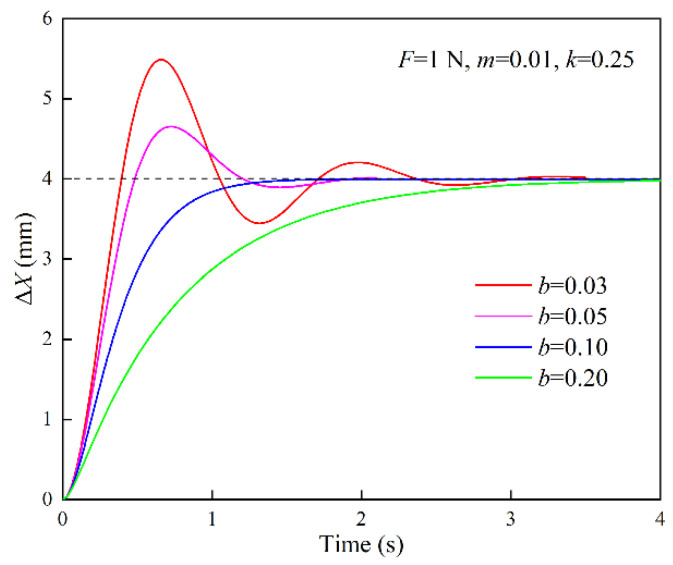
Response curves of position correction amount under different damping parameters (*m*: N·s^2^/mm; *b*: N·s/mm; *k*: N/mm).

The response curves of position correction amount under different inertia parameters are shown in Figure 4. The simulation parameters are set to {*F* = 1 N, *b* = 0.10 N·s/mm, *k* = 0.25 N/mm}. When *m* = 0.001 N·s^2^/mm, *ξ* > 1, the system is in the overdamped state; when *m* = 0.01 N·s^2^/mm, *ξ* = 1, the system is in the critically damped state; when *m* = 0.02, 0.03 N·s^2^/mm, *ξ* < 1, the system is in the underdamped state. The response curves of the position correction amount under different damping parameters are shown in Figure 5. The simulation parameters are set to {*F* = 1 N, *m* = 0.01 N·s^2^/mm, *k* = 0.25 N/mm}. When *b* = 0.20 N·s/mm, *ξ* > 1, the system is in the overdamped state; when *b* = 0.10 N·s/mm, *ξ* = 1, the system is in the critically damped state; when *b* = 0.03, 0.05 N·s/mm, *ξ* < 1, the system is in the underdamped state. When the system is in the overdamped or critically damped state, the response curve has no overshoot and oscillation, and the rise time and settling time of the critically damped system are shorter than those of the overdamped system. When the system is in the underdamped state, as the damping ratio decreases, the overshoot increases and the settling time becomes longer.

**Figure 6:**
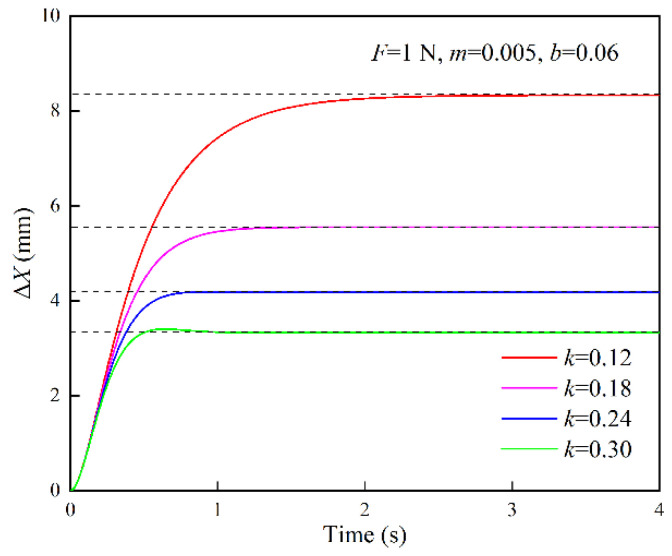
Response curves of position correction amount under different stiffness parameters (*m*: N·s^2^/mm; *b*: N·s/mm; *k*: N/mm).

The response curves of the position correction amount under different stiffness parameters are shown in Figure 6. The simulation parameters are set to {*F* = 1 N, *m* = 0.005 N·s^2^/mm, *b* = 0.06 N·s/mm}. When *k* = 0.12 N/mm, *ξ* > 1, the system is in the overdamped state; when *k* = 0.18 N/mm, *ξ* = 1, the system is in the critically damped state; when *k* = 0.24, 0.30 N/mm, *ξ* < 1, the system is in the underdamped state. With the change of the stiffness parameter, it is found that the steady-state value of the response curve changes significantly. As the stiffness parameter increases, the steady-state value decreases, that is, the position correction amount becomes smaller, thus the robot’s compliance worsens.

Through the above analysis, applying the impedance control model to the passive training of the rehabilitation robot is to improve the compliance of the rehabilitation robot and achieve the purpose of protecting the patient. The response curve needs to show no overshoot and no oscillation. In addition, the settling time should be shortened as much as possible. Therefore, the impedance model parameters should be set to the critically damped state. Since the steady-state value of the position correction amount is only affected by the stiffness parameter, the stiffness parameter can be reduced to increase the robot’s compliance.

### 4.2. Impedance Control Strategy Simulation

When simulating the passive training impedance control strategy, it is necessary to add the impedance control model on the basis of the previous position control simulation. In the simulation environment, the man-machine contact force is set to be:

(9) $$\{\begin{array}{l} F_{y}=\sin t+\sin2t+\sin4t\\ F_{z}=\cos t+\cos2t+\cos4t \end{array}$$

where, *F_y_* and *F_z_* are the components of the man-machine contact force in the *Y*-direction and *Z*-direction, respectively.

**Figure 7:**
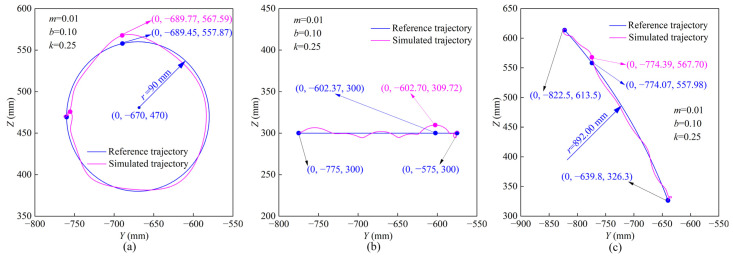
Reference trajectories and simulated trajectories under different training modes: (**a**) MOTOmed training; (**b**) CPM training; (**c**) SLR training; (*m*: N·s^2^/mm; *b*: N·s/mm; *k*: N/mm).

In the MOTOmed training mode, the reference trajectory of the robot end effector is a circular trajectory. The reference trajectory parameters are set to {the center coordinates (*x*_0_, *y*_0_, *z*_0_) = (0, −670, 470) and the radius *r* = 90.00 mm}. The impedance model parameters are selected from a set of parameters in the critically damped state: {*m* = 0.01 N·s^2^/mm, *b* = 0.10 N·s/mm, *k* = 0.25 N/mm}. The comparison between the reference trajectory and the simulated trajectory of MOTOmed training is shown in Figure 7a. It can be seen that under the action of the man-machine contact forces *F_y_* and *F*_z_, the simulated trajectory has a certain degree of offset compared with the reference trajectory, the coordinate where the maximum position offset occurs is (0, −689.77, 567.59) and the maximum offset is 9.73 mm (*Y*-direction: −0.32 mm, *Z*-direction: 9.72 mm). In the CPM training mode, the reference trajectory of the robot end effector is a beeline trajectory, and the coordinates of the starting point and the end point are set to be (0, −575, 300) and (0, −775, 300), respectively. The impedance control parameters and the contact force function are the same as those of the circular trajectory. The comparison between the CPM training reference trajectory and the simulated trajectory is shown in Figure 7b. Compared with the reference trajectory, the coordinate of the maximum position offset on the simulated trajectory is (0, −602.70, 309.72) and the maximum offset is 9.73 mm (*Y*-direction: −0.33 mm, *Z*-direction: 9.72 mm). In the SLR training mode, the reference trajectory of the robot is an arc trajectory, the coordinate of the starting point of the reference trajectory is (*x*_0_, *y*_0_, *z*_0_) = (0, −822.5, 613.5), and the coordinate of the end point is (*x*_0_, *y*_0_, *z*_0_) = (0, −639.8, 326.3), the radius *r* = 892.00 mm. The comparison between the SLR training reference trajectory and the simulated trajectory is shown in Figure 7c. Compared with the reference trajectory, the coordinate of the maximum position offset of the simulated trajectory is (0, −774.39, 567.70), and the maximum offset is 9.73 mm (*Y*-direction: −0.32 mm, *Z*-direction: 9.72 mm). From the above analysis, it is found that in the three training modes, the maximum offset values of the simulated trajectories are the same, which is related to the same settings of man-machine contact force and impedance control parameters in the simulation.

**Figure 8:**
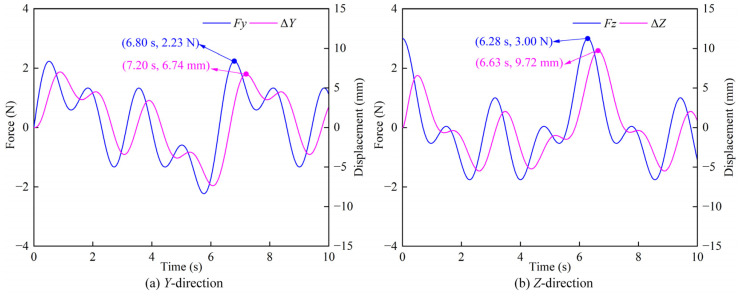
The comparison of the contact force and the position correction amount in different directions (**a**) *Y*-direction; (**b**) *Z*-direction.

The contact force and position correction amount in *Y*-direction are shown in Figure 8a. It can be seen that within the simulation time of 0–10 s, the *Y*-direction contact force fluctuates within a certain range, and at the time of 6.80 s, the contact force reaches the maximum value of 2.23 N. The fluctuation trend of the position correction amount in the *Y*-direction is consistent with that of the contact force, but there is a certain delay between the position correction amount and the contact force. At the moment of 7.20 s, the position correction amount reaches the maximum value of 6.74 mm. The contact force and position correction amount in the *Z*-direction are shown in Figure 8b. Within the simulation time of 0–10 s, the position correction amount lags behind the contact force. At the time of 6.28 s, the contact force in the *Z*-direction reaches the maximum value of 3.00 N. At the moment of 6.63 s, the *Z*-direction position correction amount achieves the maximum value of 9.72 mm. By comprehensive analysis of the above results, the time at which the maximum position offset occurs is 6.63 s in the three training modes. The maximum offsets in the three training modes are the same, indicating that the position offset is determined by the man-machine contact force and not affected by the training mode. Through the above simulations of MOTOmed training, CPM training, and SLR training, it can be shown that under the action of man-machine contact force, the rehabilitation robot shows a certain compliance by generating the position correction amount to adapt to changes of the man-machine contact force.

## 5. Experimental Verification

### 5.1. Robot Prototype and Control System

**Figure 9:**
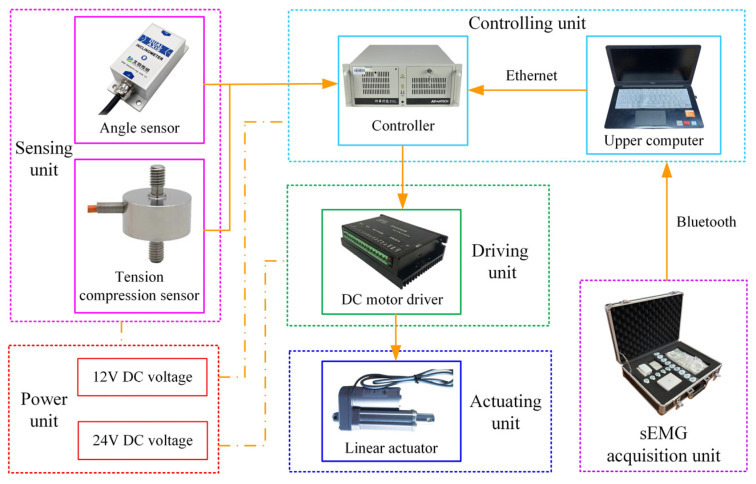
Frame diagram of lower limb rehabilitation robot control system.

The control system of the lower limb rehabilitation robot consists of the controlling unit, the driving unit, the actuating unit, the sensing unit, the sEMG acquisition unit, and the power unit, as shown in Figure 9.

The biosignal acquisition tool (PLUX wireless biosignals S.A., Biosignals Researcher, Lisbon, Portugal) collects sEMG signals in real-time through electromyography electrodes pasted on the target muscle groups of the lower limbs and transmits the signals to the upper computer (DELL Technologies Co., Ltd., Vostro 5370, Round Rock, TX, USA) through Bluetooth. Filter processing and feature value calculation are carried out within the set time period, and the feature value is transmitted to the controller through the Ethernet. The industrial controller (Advantech Technology Co., Ltd., IPC610, Suzhou, China) is used as the controller. In addition to receiving instructions from the upper computer in real-time, it can also receive signals from the tension compression sensor (HY chuangan Technologies Co., Ltd., HYLY-019, Bengbu, China) and the angle sensor (BEWIS Sensing Technologies Co., Ltd., BWK220, Wuxi, China). At the same time, the controller sends instructions to the DC motor driver (Magicon Intelligent Technologies Co., Ltd., MC-FBLD-6600, Shenzhen, China), and drives the linear actuators (Suzhou Yuancheng mingchuang Electromechanical Equipment Co., Ltd, LEC606, Suzhou, China) to perform telescopic movement. The linear actuator has a built-in incremental encoder, which can record the motion position of the DC motor to facilitate the position-based closed-loop control of the linear actuator. Angle sensors, tension compression sensors, and DC motor drivers require 12 V or 24 V DC voltage, which is provided by the power unit.

**Figure 10:**
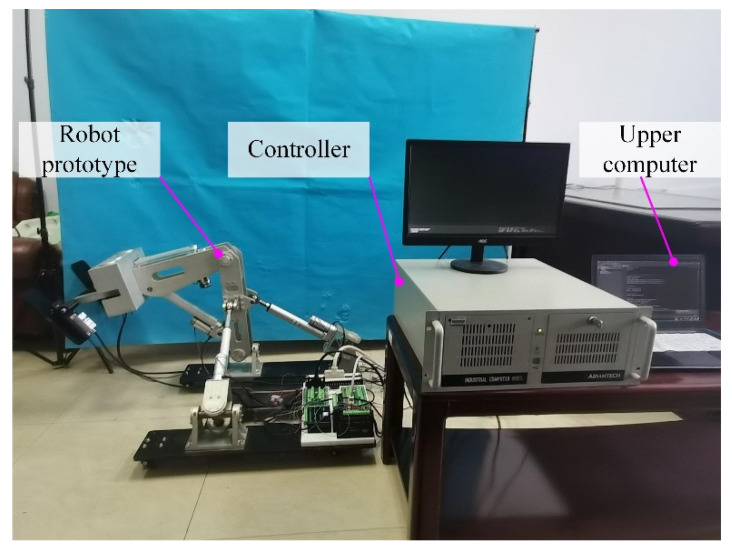
Prototype of the HE-LRR and the control system.

The prototype of HE-LRR was manufactured and integrated with the control system, which is shown in Figure 10. Universal casters with brakes are installed at the bottom of the base frame to facilitate the movement of the robot and improve the stability during rehabilitation training. The patient’s feet are placed on the foot pedal to carry out the rehabilitation training. During the implementation of this study, five healthy participants (age: 24–31 years old; height: 1670–1870 mm; thigh length: 405–455 mm; calf length: 385–420 mm) were recruited to take part in the experiment following the procedures for healthy participants as approved by the China Rehabilitation Research Center (CRRC-IEC-RF-SC-005-01), and the basic information of the participants is listed in Table 1. There were no known muscular or neurological disorders among the healthy participants. All participants completed the experimental protocol safely and reported no physical discomfort.

**Figure 11:**
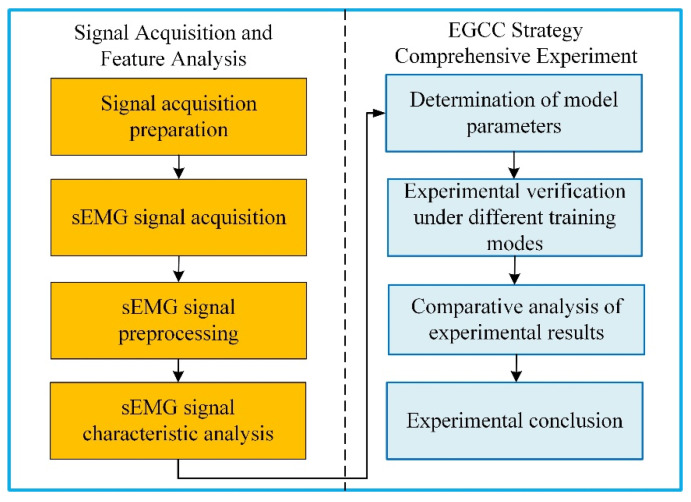
Flowchart of the experimental procedure.

The experimental procedure is shown in Figure 11. In the subsection of Signal Acquisition and Feature Analysis, the experimental processes include signal acquisition preparation, signal acquisition, signal preprocessing. and signal characteristic analysis. In the subsection of EGCC Strategy Comprehensive Experiment, the research is carried out in the order of the determination of model parameters, experimental verification, comparative analysis of experimental results. and experimental conclusion.

### 5.2. Signal Acquisition and Feature Analysis

**Figure 12:**
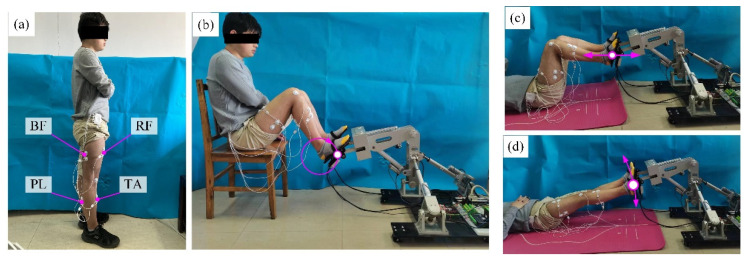
(**a**) Target muscle groups and sensor sticking positions; (**b**) MOTOmed training mode; (**c**) CPM training mode; (**d**) SLR training mode.

Before the sEMG signal acquisition experiment, the biceps femoris (BF), rectus femoris (RF), tibialis anterior (TA), and peroneus longus (PL) were selected as the target muscle groups of the lower limbs, and the surface electrodes were pasted on the corresponding skin positions of the muscle groups. The positions of the four target muscle groups of the lower limbs and the sensor sticking positions are shown in Figure 12. In the sEMG signal acquisition process, the subjects were given instructions to keep their lower limbs relaxed and not to contract their muscles actively. Their feet followed the robot end effector to move in space. Each subject participated in 12 groups of experiments for each training mode (MOTOmed, CPM or SLR). Impedance parameter settings in the 12 groups of experiments are shown in Table 2. In each group of experiments, the subjects performed 10 cycles of training.

**Figure 13:**
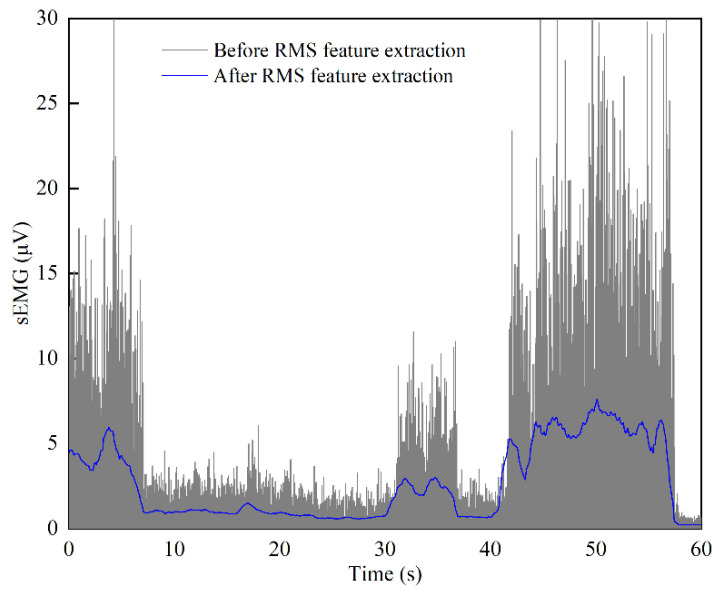
sEMG signals before and after RMS feature extraction.

The sampling frequency of the sEMG acquisition unit is 1000 Hz, and the sampling period is 1 ms. The collected original sEMG signals are in the range of 0–10 µV. After passing through the band-pass filter with a passband of 10–500 Hz, the feature value is extracted from the filtered sEMG signal and the RMS feature value is used for the time-domain quantitative analysis of the sEMG signal. Figure 13 shows the sEMG signals before and after RMS feature extraction. It can be seen that the signal characteristic of violent fluctuations is eliminated after RMS feature extraction. At the same time, the sEMG signal after the RMS feature extraction can well reflect the change trend of the original signal (before RMS feature extraction) and shows good regularity and stability. The maximum RMS values of the sEMG signal of the subjects in different training modes are extracted and statistical analysis is carried out to obtain the average value and standard deviation.

**Figure 14:**
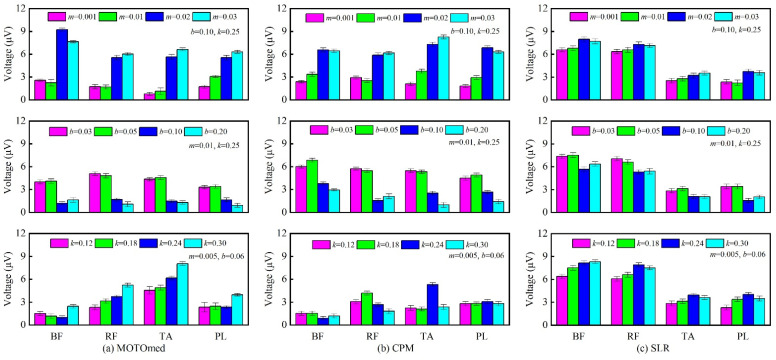
Maximum RMS value of sEMG signal in different training modes (**a**) MOTOmed mode; (**b**) CPM mode; (**c**) SLR mode (*m*: N·s^2^/mm; *b*: N·s/mm; *k*: N/mm).

Figure 14 displays the maximum RMS values of the sEMG signal in different training modes. In the MOTOmed training mode, under the condition of different impedance control parameters, the maximum RMS values of the four muscle groups are shown in Figure 14a. It can be seen that when the damping parameter and stiffness parameter are fixed values (*b* = 0.10 N·s/mm, *k* = 0.25 N/mm), the RMS values of the four muscles are at a higher level under the underdamped state (*m* = 0.02, 0.03 N·s^2^/mm) and the RMS values of the four muscles are at a lower level when under the overdamped or critically damped state (*m* = 0.001, 0.01 N·s^2^/mm). Similarly, when the inertia parameter and stiffness parameter are fixed values (*m* = 0.01 N·s^2^/mm, *k* = 0.25 N/mm), the four muscles obtain relatively high RMS values of the sEMG signals in the underdamped state. When the inertia parameter and damping parameter are fixed values (*m* = 0.005 N·s^2^/mm, *b* = 0.06 N·s/mm), the maximum RMS values of the muscle groups except for the PL muscle increase with the increase of the stiffness parameter. This is because when the stiffness parameter increases, the offset degree of the robot in response to the action of the man-machine contact force decreases, and the compliance of the HE-LRR robot is reduced, resulting in the situation where the muscle activation level cannot be released and maintained at a high level.

In the CPM training mode, under different impedance control parameters, the maximum RMS values of the four muscle groups are shown in Figure 14b. It can be seen that different muscles can obtain higher RMS values in the underdamped state, which is similar to the MOTOmed training mode. The difference is that the maximum RMS value is 8.26 ± 0.25 μV (TA muscle) in the CPM training mode, while the maximum RMS value is 9.24 ± 0.23 μV (BF muscle) in the MOTOmed training mode. In the SLR training mode, under different impedance control parameters, the maximum RMS values of the four muscles are shown in Figure 14c. It can be seen that, when *m* = 0.005 N·s^2^/mm, *b* = 0.06 N·s/mm, *k* = 0.30 N/mm, the maximum RMS value of the sEMG signal is 8.30 ± 0.24 μV (BF muscle), and when *m* = 0.005 N·s^2^/mm, *b* = 0.06 N·s/mm, *k* = 0.24 N/mm, the maximum RMS value is 7.89 ± 0.28 μV (RF muscle).

Comprehensive analysis, when the participants participate in the three training modes in a relaxed state, the RMS range of sEMG for target muscle groups is 0–9.24 μV in MOTOmed training, the RMS range of sEMG is 0–8.26 μV in CPM training, and the RMS range of sEMG is 0–8.30 μV in the SLR training (since the minimum values of RMS of different subjects were close to zero, here the lower bound value of the RMS range is determined to be zero). In the MOTOmed training mode, *RMS*_min_ and *RMS*_max_ are determined as 0 μV and 9.24 μV; in the CPM training mode, *RMS*_min_ and *RMS*_max_ are determined as 0 μV and 8.26 μV; in the SLR training mode, *RMS*_min_ and *RMS*_max_ are determined as 0 μV and 8.30 μV. The feature analysis results show that there exists a difference in the RMS range under different training modes, which proves that adopting different training modes can carry out targeted rehabilitation training for different muscle groups, so as to achieve a better effect of lower limb rehabilitation training. In particular, after RMS feature extraction, the regularity and stability of the sEMG signals are further improved, which can meet the needs of the EGCC strategy. Moreover, taking the maximum RMS values in this subsection as the reference values for normalization processing can improve the generalization ability of the EGCC strategy.

### 5.3. EGCC Strategy Comprehensive Experiment

In order to verify the control effect of EGCC strategy, validation experiments were carried out under different training modes. The participants kept their lower limbs in a relaxed state during the training process. After normalization processing, the normalized sEMG threshold was set at 0.50, 0.75, and 1.00, respectively. The inertia parameter *m*, damping parameter *b*, and stiffness parameter *k* in the EGCC strategy were set at 0.01 N·s^2^/mm, 0.10 N·s/mm, and 0.25 N/mm, respectively. For the convenience of comparison, the coefficient *a* was set to be 5 in the following EGCC strategy comprehensive experiment.

**Figure 15:**
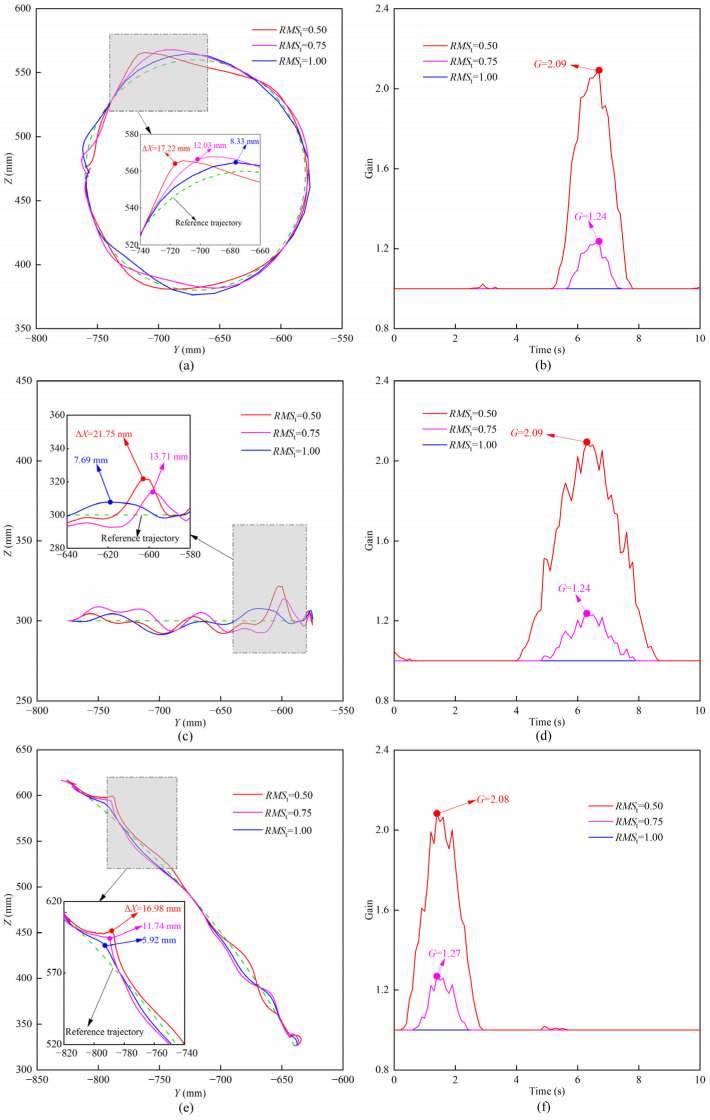
EGCC experimental results of HE-LRR. (**a**) Actual trajectories in the MOTOmed training mode; (**b**) gain parameters in the MOTOmed training mode; (**c**) actual trajectories in the CPM training mode; (**d**) gain parameters in the CPM training mode; (**e**) actual trajectories in the SLR training mode; (**f**) gain parameters in the SLR training mode.

The experimental results of the actual end trajectory and gain parameter of the lower limb rehabilitation robot under different training modes are shown in Figure 15. It can be seen that in the MOTOmed training mode, the actual trajectories in the three groups of experiments deviate to a certain extent compared with the reference trajectory (Figure 15a). When the normalized sEMG threshold is 0.50, 0.75, and 1.00, the maximum values of the position correction amount are 17.22 mm, 12.03 mm, and 8.33 mm, respectively. As can be seen from Figure 15b, when the normalized sEMG thresholds are 0.50 and 0.75, the gain parameter fluctuates locally. When the normalized sEMG threshold is 0.50, the maximum value of the gain parameter is 2.09. When the normalized sEMG threshold is 0.75, the maximum value of the gain parameter is 1.24, which shows that the decrease of the normalized sEMG threshold is beneficial in improving the compliance of HE-LRR.

In the CPM training mode, when the normalized sEMG thresholds are 0.50, 0.75, and 1.00, the maximum values of the position correction amount are 21.75 mm, 13.71 mm, and 7.69 mm, respectively, while the maximum values of the gain parameter are 2.09, 1.24, and 1.00, respectively. In the SLR training mode, when the normalized sEMG thresholds are 0.50, 0.75, and 1.00, the maximum values of the position correction amount are 16.98 mm, 11.74 mm, and 5.92 mm, and the maximum values of the gain parameter are 2.08, 1.27, and 1.00, respectively. Comparing the results in the three training modes, although the maximum values of the position correction amount are different, the gain parameters are relatively close to each other. This is because when the normalized sEMG thresholds are 0.50, 0.75, and 1.00, the gain parameters have a maximum value of 2.25, 1.3125, and 1.00, respectively, which enables the position correction amount of the lower limb rehabilitation robot to be maintained within a certain range to prevent secondary damage caused by excessive offset.

In addition, it can be seen from Figure 15b,d,f that the gain parameter is larger than 1.00 in a relatively short time. Since there is a clear functional relationship between the gain parameter and normalized sEMG threshold, it shows that the normalized sEMG can recover below the threshold in a short time. This is due to the fact that as the gain parameter increases, the position offset occurring in the direction of the man-machine contact force increases and the compliance of the lower limb rehabilitation robot is enhanced, which is conducive to the recovery of muscle activation. When the normalized sEMG threshold is set as 1.00, the EGCC strategy can be used to identify abnormal sEMG signals and increase the compliance of the lower limb rehabilitation robot to protect the participant. In conclusion, the EGCC strategy can play a significant role in regulating the compliance of the lower limb rehabilitation robot and increasing the safety of the participant.

## 6. Conclusions and Future Work

Aiming at the problems of insufficient physiological information and unsatisfactory safety performance in the existing compliance control strategies for the lower limb rehabilitation robot during passive training, this paper developed an sEMG-based gain-tuned compliance control strategy and carried out simulation and experimental research based on this control strategy. The main conclusions are as follows:

(1) The EGCC strategy without sEMG information was simulated and analyzed. The influence of impedance control parameters on the position correction amount of the robot end effector was studied through simulation, and the change rules of the robot end trajectory, man-machine contact force and position correction amount analyzed, providing a basis for establishing a gain-tuned control strategy fusing the sEMG information.
(2) The experimental acquisition and feature analysis of sEMG signals were carried out to determine the influence of impedance control parameters on the RMS values of sEMG under different training modes and the normal range of RMS values. The preprocessed sEMG has good regularity and stability, which can provide a reference for the normalization processing of sEMG signals in the EGCC strategy.
(3) Based on the lower limb rehabilitation robot control system, the control effect of EGCC strategy was studied in different training modes. The influences of the normalized EMG threshold on the robot’s end trajectory and the gain parameter were analyzed. The results prove that EGCC strategy can play a significant role in improving the compliance and safety of the lower limb rehabilitation robot, which validates the rationality of the EGCC strategy.

Although the control strategy in this paper was verified in the end-effector robot system, the basic methodology can also be applied in the exoskeleton lower limb robot system. There are still some shortcomings in the current research work, for example, the simulation and experimental research of the EGCC strategy were mainly carried out in three training modes: MOTOmed, CPM, and SLR, and the normalized sEMG threshold was required to be set manually. Future research work will be committed to solving the problems of the EGCC strategy validation in various training modes as well as the autonomous learning and optimization of the EGCC strategy model.

## Figures and Tables

**Table 1:** Basic information of the participants in the experiments.

Number	Age (year)	Height (mm)	Thigh Length (mm)	Calf Length (mm)
1	31	1790	430	405
2	28	1720	430	400
3	24	1870	455	420
4	30	1670	405	385
5	28	1690	415	400

**Table 2:** Impedance parameter setting in the 12 groups of experiments.

Group Number	Inertia Parameter (N·s^2^/mm)	Damping Parameter (N·s/mm)	Stiffness Parameter (N/mm)
1	0.001	0.10	0.25
2	0.01	0.10	0.25
3	0.02	0.10	0.25
4	0.03	0.10	0.25
5	0.01	0.03	0.25
6	0.01	0.05	0.25
7	0.01	0.10	0.25
8	0.01	0.20	0.25
9	0.005	0.06	0.12
10	0.005	0.06	0.18
11	0.005	0.06	0.24
12	0.005	0.06	0.30

## Data Availability

The original data contributions presented in the study are included in the article; further inquiries can be directed to the corresponding authors.

## Abbreviations

EGCC	Electromyography-based gain-tuned compliance control
sEMG	Surface electromyography
CPM	Continuous passive motion
SLR	Straight leg raise
SVM	Support vector machine
HE-LRR	Hybrid end-effector lower limb rehabilitation robot
BF	Biceps femoris
RF	Rectus femoris
TA	Tibialis anterior
PL	Peroneus longus
RMS	Root mean square
